# No re-calibration required? Stability of a bioelectrochemical sensor for biodegradable organic matter over 800 days

**DOI:** 10.1016/j.bios.2021.113392

**Published:** 2021-10-15

**Authors:** Martin WA. Spurr, Eileen H. Yu, Keith Scott, Ian M. Head

**Affiliations:** a School of Natural and Environmental Sciences, Newcastle University, Newcastle Upon Tyne, UK; b Department of Chemical Engineering, Loughborough University, Loughborough, UK; c School of Engineering, Newcastle University, Newcastle Upon Tyne, UK

**Keywords:** Microbial fuel cell, Calibration, Resistance, Substrate

## Abstract

Microbial Fuel Cells (MFCs) operated as biosensors could potentially enable truly low-cost, real-time monitoring of organic loading in wastewaters. The current generated by MFCs has been correlated with conventional measures of organic load such as Biochemical Oxygen Demand (BOD), but much remains to be established in terms of the reliability and applicability of such sensors. In this study, batch-mode and multi-stage, flow-mode MFCs were operated for over 800 days and regularly re-calibrated with synthetic wastewater containing glucose and glutamic acid (GGA). BOD_5_ calibration curves were obtained by normalising the current measured as a percentage of maximum current. There was little drift between recalibrations and non-linear Hill models of the combined dataset had *R*^2^ of 88–95%, exhibiting a stable response over time and across devices. Nonetheless, factors which do affect calibration were also assessed. Increasing external resistance (from 43.5 to 5100 Ω) above the internal resistance determined by polarisation curve decreased the calibration upper limit from 240 to 30 mg/l O_2_ BOD_5_. Furthermore, more fermentable carbon sources increased the detection range, as tested with samples of real wastewater and synthetic media containing GGA, glucose-only and glutamic acid-only. Biofilm acclimatisation therefore did not account for differences between aerobic oxygen demand determinations and anaerobic MFC responses; these are likely attributable to competitive processes such as fermentation. This further highlights the potential for MFCs as real-time sensors for organic load monitoring and process control in addition to BOD-compliant measurement systems.

## Highlights

• The calibration of a bioelectrochemical sensor was assessed over >800 days and remained stable with independent replicates.
• The dynamic biochemical oxygen demand detection range was increased as external resistance was lowered.
• Analyte composition affected sensor BOD_5_ prediction thus calibrants should be similar in composition to the analyte measured.

## 1 Introduction

Determination of water quality is important for treatment and security of downstream ecosystems. A crucial parameter determining water quality is Biochemical Oxygen Demand (BOD); the dissolved oxygen required by micro-organisms to oxidise organic matter present in the water. BOD is conventionally measured by offline, five-day tests (BOD_5_; APHA, 1999) or online with expensive transducers requiring frequent re-calibration and maintenance (Jouanneau et al., 2013).

The electrical outputs from Bioelectrochemical Systems (BES), including Microbial Fuel Cells (MFCs), have been correlated to the concentration of biodegradable organic carbon present with high accuracy (Gupta et al., 2019; Sonawane et al., 2020). There is potential to operate sensors with minimal maintenance over multiple years (Kim et al., 2003a), either in batch-fed mode as an offline testing platform or for continuous process monitoring. To prevail against other online BOD sensors (where unreliability has limited their uptake), the stability of the calibrated MFC response must be defined. Especially as membrane fouling or cathode degradation may necessitate recalibration (Kim et al., 2003b; Sonawane et al., 2020).

Optimum MFC operating parameters have been extensively researched for electricity generation and wastewater treatment (Zhou et al., 2013), however better understanding of parameters which affect calibration is further required (Hsieh et al., 2015). For example decreasing the hydraulic retention time has been shown to decrease the BOD_5_ detection range with multi-stage, flow-mode MFCs (Spurr et al., 2018). Most researchers have used low external resistance to prevent limitation of electron donation by the biofilm to the electrode (e.g. R_Ext_ = 10 Ω; Kang et al. (2003)). However, a systematic assessment of calibrations at different resistances has not been undertaken. Greater sensitivity has been observed operating MFCs at R_Ext_ associated with peak power for both a limited BOD_5_ range (5–50 mg/l) (Gao et al., 2021) and detecting specific toxic compounds (Yi et al., 2019). Variations in up- and down-shift response times have also been established at different R_Ext_ (Moon et al., 2004; Di Lorenzo et al., 2009; Zhang and Angelidaki, 2012). Additionally, it has been found that decreasing R_Ext_ can change the response profile from a plateau to a peak (Gil et al., 2003).

As we highlighted previously (Spurr et al., 2018), there is an absence of standardisation in reporting MFC-based BOD sensor performance. Validation methods include BOD_5_, chemical oxygen demand (COD), dissolved organic carbon (DOC) and substrate concentration; measurements which have units of mg/l but are not comparable and not all account for biodegradability of organic substrates present. A wide array of substrates have also been used for calibration including acetate, glucose, glucose and glutamic acid (GGA) and real wastewater. The assumption has been that these synthetic wastewater analogues can be interchangeably validated against the BOD_5_ test.

There have been recommendations to acclimatise MFC biofilms with substrates similar in composition to analytes (Kim et al., 2003b), as many authors have noted an under-estimate of real wastewater BOD by sensors with synthetic calibrants (Kang et al., 2003; Moon et al., 2005; Di Lorenzo et al., 2009). Feng et al. (2013) proposed a model to identify four different substrates present at the same concentration by the peak height and area of the current response profile. Kaur et al. (2013) reported different responses for MFCs acclimated with different volatile fatty acids. Hsieh et al. (2015) tested various carbohydrates, amino acids, organic acids and alcohols at a fixed concentration of 100 mg/l O_2_ BOD_5_ with GGA-acclimatised MFCs and established different responses with each. By contrast, Zhang and Angelidaki (2011) and Yang et al. (2013) stated that their synthetic calibrations (with acetate/glucose and GGA respectively) could successfully predict real wastewater BOD_5_ values with high accuracy.

The limited reports of testing with real wastewaters indicate the need for further investigation. ElMekawy et al. (2018) reviewed analytical applications of MFCs and deduced that improvements to stability, repeatability and sensitivity are needed for commercial viability. As we have described previously (Spurr et al., 2018), a multi-stage MFC configuration possesses advantages in terms of extending the BOD detection range beyond the capacity/saturation limit of a single MFC and thus extends the potential application to higher strength wastewaters. The ordered response of each staged MFC can further indicate inhibition by toxicity (Spurr et al., 2020; Godain et al., 2020) and therefore potentially different substrate responses as well.

The aim of this study was to determine the stability of MFC sensor calibrations under fixed conditions over long-term operation, this was achieved by repeated calibration over 800 days with batch- and flow-mode MFCs. Further we sought to improve the understanding of factors which do affect calibration range, namely R_Ext_ and organic carbon source. MFC outputs were calibrated with a range of BOD concentrations to saturation at a range of external resistances, with synthetic media containing glucose, glutamic acid and a 1:1 GGA mixture and tested with real samples of municipal wastewater.

## 2 Material and methods

### 2.1 Calibration & feed media

#### 2.1.1 Synthetic wastewater media

MFCs were operated with phosphate-buffered synthetic medium containing 1:1 (w/w) glucose and glutamic acid (GGA; as in Spurr et al., 2018). Furthermore, in this study some media were prepared using solely glucose (G) or glutamic acid (GA) carbon sources. To alter organic loading the carbon source concentration (G, GA or GGA) was varied between 25 and 2000 mg/l. The justification for using GGA as the primary carbon source for calibration was to permit comparisons with the standard organic source of the 5-day BOD test (APHA, 1999), and also with approximately a third of reported studies on MFC BOD sensors which used glucose/GGA-based media (as reviewed in Spurr et al. (2018)).

#### 2.1.2 Raw wastewater

Activated sludge inocula and raw wastewater were collected from Tudhoe Mill sewage treatment works (Northumbrian Water). To enable comparisons at equal conductivities with synthetic media, the wastewater conductivity was increased (from approximately 4 to 8 mS/cm) by titrating 2 mol/dm^3^ potassium phosphate buffer solution.

### 2.2 Microbial Fuel Cells (MFCs)

All MFCs and open circuit potential (OCP; non-polarised) control reactors were operated on an aluminium heating platform at 28.2 ± 2.0 °C, to minimise variations due to ambient temperature. MFCs were operated with external resistances (R_Ext_) of 43.2, 305, 953 or 5100 Ω. MFC cell voltages (*V*) were recorded with a NI-USB 6225 datalogger and LabVIEW SignalExpress data acquisition software (National Instruments). Current density was calculated as *I* = *V*/(*R*_Ext_.*A*_Eff_) where A_Eff_ is the effective electrode area not concealed by a gasket.

#### 2.2.1 Batch-mode MFCs

50 ml single-chamber MFCs (Figure S1, Electronic Supplementary Information; ESI) contained carbon cloth anodes and 0.5 mg/cm^2^ Pt gas diffusion cathodes (both 12.6 cm^2^ A_Eff_) separated by Fumapem F-930 cation exchange membranes (FuMA-Tech). Following each batch the liquid contents of the anodic chamber (anolyte) were removed by syringe and refilled with fresh medium.

Duplicate 50 ml batch-mode MFCs (termed MFCs A and B) were inoculated with activated sludge and operated with synthetic medium for 848 days (Figure S2, ESI). On the penultimate day of operation, independent batch-mode MFCs (MFCs C and D) were inoculated with MFC A and B effluent and operated for 183 days (Table 1; Figure S3, ESI).

**Table 1:** Details of experiments regarding operation mode, BOD calibration, external resistance (R_Ext_) effect, substrate effect and 16S rRNA gene sequencing performed with MFCs in the present study.

MFCs	Mode	BOD	R_Ext_	Substrate	16S
		calib.	effect	effect	seq.
A&B	Batch	*✓*	*✓*	*✓*	*✓*
C&D	Batch	*✓*		*✓*	
OCP A,B,C	Batch				*✓*
1A,1B,1C	Flow	*✓*			†
2A,2B,2C	Flow	*✓*			†
3A,3B,3C	Flow	*✓*			†
1D,1E,1F	Flow	*✓*		*✓*	
2D,2E,2F	Flow	*✓*		*✓*	
3D,3E,3F	Flow	*✓*		*✓*	

†Community data presented in Spurr et al. (2018).

#### 2.2.2 OCP electrodes

To determine microbial colonisation of non-polarised electrodes and act as a control to compare with the batch-mode cells, triplicate 11.0 cm^2^ carbon cloth electrodes were placed into 50 ml sealed vessels and maintained at OCP (OCP A, B and C). Vessels were inoculated with combined effluent from MFCs A and B (from day 812) and medium replacement was done in-line with batch-mode MFC feeding.

#### 2.2.3 Single-pass, continuous system for flow-mode MFCs

10 ml single-chamber MFCs (Figure S4a, ESI) were constructed using the same materials as the batch-mode MFCs with 4.91 cm^2^ A_Eff_. In brief, each replicate feed line comprised a medium bottle containing ¡5600 ml medium which was pumped at 1.24 ml/min by a peristaltic pump in continuous, single-pass mode into a sterile drip chamber, past a UV lamp (to prevent upstream contamination) to a cascade of three-stage, hydraulically connected MFCs and towards a waste vessel (Figure S4b, ESI).

As previously reported (Spurr et al., 2018), triplicate three-stage arrays of MFCs were operated as BOD sensors for 757 days (referenced here as the ‘ABC’ series; Figure S5, ESI). The nomenclature is thus; in triplicate feed lines (A, B, C) the MFCs 1A, 1B, 1C were in the first stage, MFCs 2A, 2B, 2C in the second stage and MFCs 3A, 3B, 3C in the third stage. In another study (Spurr et al., 2020), an independent triplicate set of three-stage MFCs was operated over 148 days (termed here ‘DEF’ series; Figure S6, ESI). Both MFC series were inoculated with effluent from batch-mode MFCs A and B at different time points and comparative experiments are evaluated in the present study (Table 1).

### 2.3 Analytical methods

BOD_5_ tests were done according to standard protocol (APHA, 1999). Dissolved oxygen measurements were taken from four replicate anolyte samples comprising a single initial reading and three final readings after 5 days incubation at 20 °C. Conversion ratios of 0.632 BOD_5_/G, 0.600 BOD_5_/GGA and 0.577 BOD_5_/GA were experimentally determined to estimate BOD_5_ values for media with defined composition.

COD was determined with a potassium dichromate assay refluxed at 2 h at 148 °C and measuring absorbance at 605 nm with a Spectroquant Pharo 300 spectrophotometer (Merck Millipore). DOC was measured in 0.2 *μ*m-filtered samples with a Total Organic Carbon analyser (TOC-5050A; Shimadzu). Anion concentrations were measured using an ion chromatography system (ICS-1000 with AS14A column; Thermo Fisher Scientific). pH and conductivity were measured using HI-9025 (Hanna Instruments) and FE30 (Mettler Toledo) meters respectively.

MFC performance was assessed from polarisation/power density curves measured from OCP and connecting sequentially lower R_Ext_ (52300–10.6 Ω; Section S1, ESI).

### 2.4 Biochemical oxygen demand calibration & current density normalisation

Data treatments were done according to Spurr et al. (2018). MFC current densities were normalised (0–100% of *I*_*Max*_) to permit comparisons between cells of different electrochemical performance (i.e. due to cathode degradation). Average stable current density ($\bar{I}$) from flow-mode MFCs was defined in the period in each cycle that *dI*/*dt* (in *μ*Acm^−2^ hour^−1^) was below 3% of the peak current density.

Calibration curves of current density (*y* in *μ*A/cm^2^ or normalised %) versus BOD_5_ (*x* in mg/l O_2_) were fitted with linear (Equation (1)) and Hill (Equation (2)) models;

(1) y=mx+c

(2) $$y=\frac{v_{Max}x^{h}}{K_{M}^{h}+x^{h}}$$

where *m* is slope, *c* is y-intercept, *v*_*Max*_ is maximum current density, *K*_*M*_ is concentration at half-maximal rate and *h* is the Hill coefficient. R (R Core Team, 2020) and the ‘drc’ package (Ritz et al., 2015) were used to determine the coefficient of determination (R^2^), residual standard deviation (SD_Res_) and 95% confidence bands of fitted coefficients. Significance was determined from ANOVA linear regression models (p-value <0.05 deemed significant correlation) and lack-of-fit tests on Hill models (p-value <0.05 indicated model lacked fit).

### 2.5 Microbial community analysis

DNA was extracted from biomass on electrode materials using a PowerSoil DNA kit (Mo Bio Laboratories). Barcoded V4f and non-barcoded V5r primers with PCR master mix (MegaMix-Blue; Cambio) were used to amplify the V4–V5 region of 16S rRNA genes. PCR-amplified 16S rRNA gene fragments were purified twice with an Agencourt AMPure XP kit (Beckman Coulter). DNA was sequenced from a pooled amplicon library on an IonTorrent PGM using a Hi-Q kit (Thermo Fisher Scientific). 16S rRNA gene sequence data were analysed using the QIIME software package (Caporaso et al., 2010). Microbial communities were compared using Principle Coordinate analysis and estimates of abundance of different taxa were calculated based on total electrode cell densities corrected for 16S rRNA gene copy numbers in each taxon (Stoddard et al., 2015). Microbial community data for the flow-mode electrodes were reported previously in Spurr et al. (2018) and the batch-mode and OCP electrodes are reported here. Further protocols (including accession numbers) are in Section S2, ESI.

## 3 Results & discussion

### 3.1 Long-term calibration drift & sensor reproducibility

#### 3.1.1 Re-calibration of batch-mode MFCs

**Fig. 1:**
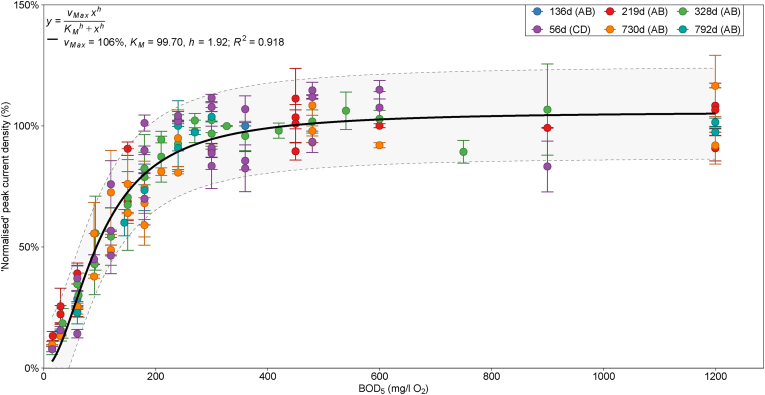
Average peak current density calibration data (normalised by maximum current density) against BOD_5_ (estimated from GGA concentration) obtained during calibrations at different time points during operation of batch-mode MFCs A, B, C and D with R_Ext_ = 43.2 Ω fitted with the Hill equation. The legend states the calibration starting day and MFC series used. The shaded band represents the 95% prediction interval from the Hill model and error bars are the range of values from duplicate cells.

To determine the effect of calibration drift and reproducibility under identical conditions (i.e. GGA medium, R_Ext_ of 43.2 Ω, 28 °C), BOD_5_ calibrations of batch-mode cells A and B initiated on days 136, 219, 328, 730 and 792 (since inoculation) were collated. Furthermore, cells C and D were calibrated under these conditions (on day 56) to establish if the response was reproducible with replicate cell architectures. Current density normalisation was necessary to make valid comparisons between calibrations (Fig. 1) as cathode potentials decreased over time (likely due to catalyst degradation or membrane fouling) and increased when fresh membrane-cathode assemblies were installed (anode electrodes were not exchanged throughout operational periods).

The modelled parameters for each normalised calibration were approximately equal within one standard deviation (Table S1, ESI). The combined data from repeated calibration of batch-mode MFCs obtained the same linear calibration range of 15–240 mg/l O_2_ BOD_5_ (Figure S7, ESI). Most points fell within the 95% prediction band of the Hill model and regression statistics of R^2^ = 0.918, SD_Res_ = 9% and lack-of-fit p-value = 0.400 indicated a statistically significant fit (Fig. 1). There was no appearance of bias through time, with as much variation within calibrations as between them. Additionally, the calibration performed using independent cells C and D was very similar to the response from cells A and B (Fig. 1; purple).

The calibration range was not significantly affected by changing performance in the MFC such as cathode degradation, membrane-electrode assembly replacement or anodic biofilm age. Therefore, effectively, the MFC sensor required no re-calibration if the maximum current density was determined regularly for subsequent data normalisation. The periodic maximum current density determination could be classed as a single-point calibration (as is done with dissolved oxygen probes in air to calibrate the 100% value for example), however significantly it does not require the lengthy preparation and validation with a range of media at different concentrations. This is an important finding as it indicates maintenance requirements can be minimal, with significant implications for future commercial viability of MFC-based BOD sensors.

#### 3.1.2 Re-calibration of flow-mode MFCs

**Fig. 2:**
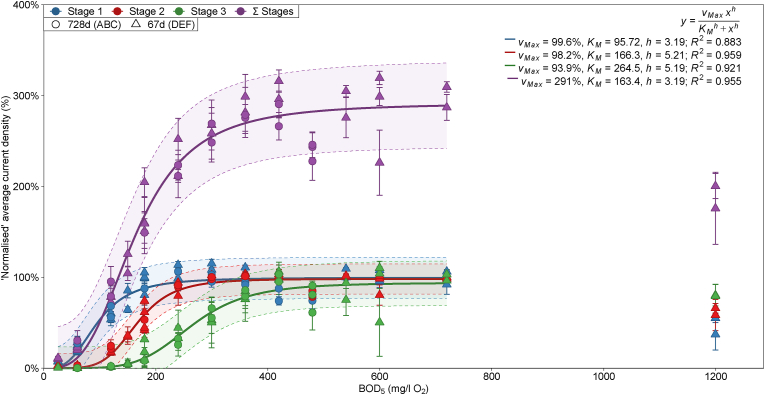
Normalised average current density calibration curves fitted with the Hill equation against BOD_5_ (estimated from GGA concentration) for data obtained during calibration of the ‘ABC’ series (circles) and ‘DEF’ series (triangles) of flow-mode MFCs. The Σ Stages data is normalised to 300% (sum of three MFC stages) to permit comparisons to non-normalised data for convenience. Shaded bands represent the 95% prediction intervals from model lines and error bars are ±SD from triplicate cells.

A calibration was performed with the triplicate, three-stage ‘ABC’ series of flow-mode MFCs after 728 days of operation. Additionally, an independent calibration was performed with the ‘DEF’ series after 67 days of operation. Both calibrations were carried out under the same conditions with a flow-rate of 1.24 ml/min and R_Ext_ of 43.2 Ω. Normalised, stable current densities were recorded after a period of stabilisation upon changing the BOD_5_ concentration at the medium bottle (Fig. 2). In all stages, current decrease due to substrate excess inhibition was observed with 1199 mg/l O_2_ BOD_5_ (2000 mg/l GGA) medium, thus these data were omitted from calibration models.

It is evident that the flow-mode cells exhibited the same stable and consistent re-calibration behaviour observed with the batch-mode cells (Section 3.1.1). The two independent flow-mode calibrations correlated, and the between-calibration variation was not more than within-calibration variation (Fig. 2). The combined calibration with normalised data had K_M_ values of 96, 166 and 265 mg/l O_2_ BOD_5_ for the first, second and third stages of MFCs respectively (within ±8 mg/l O_2_ of the values in the ‘ABC’ calibration). The later-stage MFCs responded to higher concentrations of substrate (as measured at the medium bottle/inlet), likely due to the consumption of substrate in the prior MFCs. The non-linear regression statistics were R^2^ = 0.883–0.959 and SD_Res_ = 8–22%.

The fitting of the linear regression models was statistically significant (Table S1, ESI), however the Hill model lack-of-fit p-values were significant above 450 mg/l O_2_ BOD_5_, or when the 480 and 600 mg/l O_2_ values were included. It is therefore thought this lack-of-fit pertained to data fitting in the high concentration, non-linear asymptote. In these two calibration cycles, at concentrations expected to be at saturation, current densities were less than 100% (Fig. 2), which appeared to be associated with medium cycles following periods of starvation. Cathode gas diffusion electrode degradation and/or membrane fouling during the calibration period may have also contributed as they were the final cycles performed in each series.

### 3.2 Effect of external resistance on calibration

**Fig. 3:**
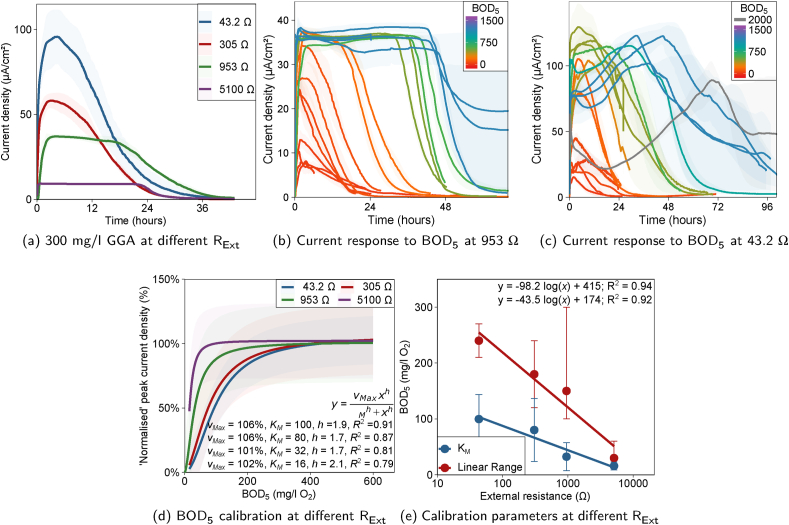
For MFCs A & B at R_Ext_ of 43.2, 305, 953 and 5100 Ω (a) the typical average current density during a 300 mg/l GGA medium cycle. Shaded bands are the range between duplicate MFCs. (b) and (c) Average current density during each medium cycle at 953 and 43.2 Ω respectively. Each average response line is coloured by the medium BOD_5_ and shaded by the range between cells. Substrate-inhibited response was observed at 43.2 Ω (c) with BOD_5_ concentrations ¿ 750 mg/l O_2_ (dark blue - grey). (d) ‘Normalised’ peak current density Hill model lines against BOD_5_ obtained from combined calibrations at each resistance. Shaded bands represent the 95% prediction interval. (e) Calibration model parameters (linear range and K_M_) plotted against the logarithm of R_Ext_ (range error bars are the next nearest calibrated BOD_5_ values within each calibration). (For interpretation of the references to colour in this figure legend, the reader is referred to the Web version of this article.)

Whilst operating batch-mode MFCs A and B the external resistance was changed periodically (Figure S2, ESI). With increasing R_Ext_ a reduction in current density was observed (Fig. 3a). This was further evidenced by the polarisation curve profiles (Figure S8, ESI) which determined a mean internal resistance (R_Int_) of 233 ± 64 Ω. Additionally, the shape of the batch cycle response changed from a peak to a plateau (Fig. 3a; 3b vs. 3c), attributed previously to external resistance becoming a rate-limiting factor (Gil et al., 2003). The response time decreased from 2.3 ± 1.6 h to 1.4 ± 1.4 h with increasing R_Ext_ of 43.2–5100 Ω (Table S2, ESI), in agreement with the findings of Moon et al. (2004).

At 953 Ω R_Ext_ a saturation plateau was reached at low concentration and subsequent increases in BOD_5_ increased cycle duration and thus coulombs passed (Fig. 3b). Negligible substrate inhibition was observed at high BOD_5_ concentrations indicating greater resilience of the anodic biofilm. Whereas, the response at 43.2 Ω exhibited a peak approximately 1 h after medium replacement up to 750 mg/l O_2_ BOD_5_, and at higher levels a second peak appeared between 24 and 72 h and the first peak reduced (Fig. 3c).

In anolyte samples, taken prior to medium replacement, COD was below the lower detection limit of the assay (30 mg/l). Consequently, substrate was assumed to be fully consumed in cycles which ran to completion (approximately 0 *μ*A/cm^2^). Coulombic efficiency decreased as R_Ext_ increased (Table S2, ESI), attributable to the biofilm oxidation rate becoming limited, resulting in less substrate consumed electrogenically and therefore greater substrate availability for competitive processes (e.g. oxygen diffusion through the membrane or fermentation).

The effect of R_Ext_ of 305, 953 and 5100 Ω on calibration was investigated in comparison to the calibrations performed at 43.2 Ω (Fig. 1 and S2, ESI). A distinct reduction in the calibration range (and therefore K_M_) was observed with increasing R_Ext_ (Fig. 3e). At 305 Ω, a similar sigmoidal-shaped calibration was recorded (Fig. 3d) with K_M_ value of 80.1 ± 56.4 mg/l O_2_ BOD_5_ compared to 99.7 ± 44.0 mg/l (43.2 Ω). This corresponded to a linear calibration range of 15–180 mg/l O_2_ BOD_5_; a reduction of 60 mg/l. As R_Ext_ was increased to 953 and 5100 Ω, calibration curves steepened with K_M_ values of 32.4 ± 25.2 and 15.8 ± 7.3 mg/l O_2_ and calibration ranges of 15–150 mg/l and 15–30 mg/l respectively (Fig. 3d). Based on the intercept of the log-linear regression lines (Fig. 3e), the theoretical maximum linear range (at short-circuit R_Ext_ = 0 Ω) was 415.3 ± 50.7 mg/l and maximum K_M_ of 174.5 ± 25.1 mg/l O_2_ BOD_5_.

This demonstrated the effect external resistance has on the calibration range. As R_Ext_ increased the BOD saturation concentration decreased resulting in a decreased calibration range. This corroborated the findings of Wu et al. (2015) for acetate-fed MFCs with a limited R_Ext_ range of 20–100 Ω. In that study the Michaelis-Menten K_M_ value decreased from 141 to 41 mg/l O_2_ COD with increasing R_Ext_. Decreasing R_Ext_ below R_Int_ did not significantly improve the calibration range, and furthermore, at too small a resistance the voltage can become noisy to measure and there may be possibility of cell voltage reversal (Ren et al., 2014).

### 3.3 Effect of substrate on calibration

#### 3.3.1 Effect of synthetic wastewater composition on calibration

Batch-mode MFCs A, B, C and D were calibrated as BOD sensors using different concentrations of solely glucose, solely glutamic acid and 1:1 GGA media (Figures S2 and S3, ESI). Substrate was alternated in the A & B calibrations (40 days), whereas with cells C & D each was calibrated in succession (Figures S9 and S10, ESI). During the lengthy calibration of cells C and D (114 days), the peak cathode potential decreased by approximately 0.4 mV per day (Figure S10, ESI).

At high concentrations of glucose (¿450 mg/l), an immediate current response was observed which peaked within 2 h, followed by a steady decline as substrate depleted. This was sometimes accompanied by a shoulder on the peak (Figure S11d, ESI), potentially associated with utilisation of acetate generated from fermentation products (e.g. propionate; Feng et al. (2013); Kaur et al. (2013)). At high concentrations of glutamic acid (Figure S11e, ESI) a broad peak was observed after approximately 24 h. In most high-concentration GGA medium cycles bimodal peaks occurred at 2h and 24h with the second peak usually exhibiting higher current density (Fig. 3c, S11f, ESI). Comparing medium cycles of 1000 mg/l it is evident that the bimodal response could be attributed to sequential glucose oxidation followed by glutamic acid oxidation (Figure S11d–f, ESI).

**Fig. 4:**
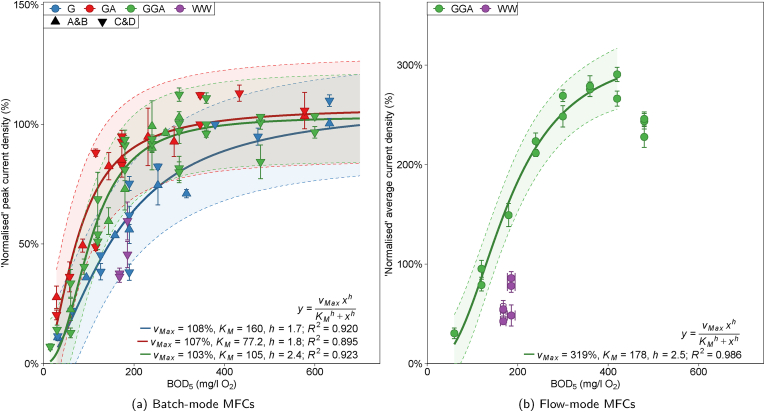
Position of real wastewater samples (purple) on Hill-modelled calibration curves of (a) ‘normalised’ peak current density from batch-mode cells A, B, C and D with different concentrations of glucose (G), glutamic acid (GA) and GGA and (b) ‘normalised’ average summed current density from multi-stage MFC cells in channels D, E and F against BOD_5_ (estimated from substrate concentration). Error bars are the range of values from duplicate cells (cell pair indicated by shape) for batch-mode calibrations and ± 1SD for triplicate flow-mode cells. The flow-mode data is normalised to 300% (sum of three MFC stages) to permit comparisons to non-normalised data for convenience. Shaded bands are the 95% prediction interval from model lines. (For interpretation of the references to colour in this figure legend, the reader is referred to the Web version of this article.)

The normalised GGA calibration curve was approximately the mean of the two single-substrate calibrations. For glucose, glutamic acid and GGA, the half maximal concentrations (K_M_) from Hill models were 160 ± 77, 77 ± 39 and 105 ± 38 mg/l O_2_ BOD_5_ respectively and the average anode potential minima were −311 ± 46, −347 ± 40 and −336 ± 25 mV vs. Ag/AgCl respectively. The glutamic acid calibration exhibited the steepest response (highest sensitivity/smallest range; Fig. 4a, GA) and lowest anode potentials indicating a lower overpotential; potentially from the bioanode utilising a different metabolic pathway to generate electricity.

There was insufficient data to reject the null hypothesis from ANOVA of single-substrate Hill models (that each single-substrate model was the same as an all-data model; p-values = 0.0643 [G] and 0.0773 [GA]). However, the significant ANOVA of linear regression (p-value = 1.24 × 10^−6^) indicated that linear, single-substrate calibrations were distinct from the GGA calibration with upper detection range limits of 253, 173 and 240 mg/l O_2_ BOD_5_ for G, GA and GGA respectively. Therefore, the sensor calibrated with a specific substrate did not accurately predict BOD_5_ values of analytes different in composition.

#### 3.3.2 Comparison of validation techniques for glucose, glutamic acid and GGA-based media

Glucose (G), glutamic acid (GA) and GGA media of 300 mg/l substrate concentration were analysed using BOD_5_ and COD assays and the theoretical oxygen demand (ThOD) and organic carbon (ThOC) were calculated. The MFC response outputs including average current density ($\hat{I}$), charge density (*Q*_*Cyc*_) and coulombic efficiency (*C*_*E*_) over a medium cycle (until depletion at approximately 24 h) were determined from batch-mode cells A, B, C and D (Table 2). With all validation methods the GGA values were approximately the mean of the glucose-only and glutamic acid-only values.

**Table 2:** Comparison of values measured by validation methods (BOD_5_ and COD) and MFC response outputs (peak current ($\hat{I}$), charge over cycle (*Q*_*Cyc*_) and coulombic efficiency (*C*_*E*_)) for a medium containing 300 mg/l glucose, glutamic acid and GGA. The MFC data are the average (±SD) from batch-mode cells A, B, C & D.

Validation Method	GGA	Glucose		Glutamic acid		*t*-test (*p*-value) †
	Value	Value	*% of GGA*	Value	*% of GGA*	
Mass conc. (mg/l)	300.0	300.0	(*100%*)	300.0	(*100%*)	–
Molar conc. (mmol/dm^3^)	1.833	1.665	(*91%*)	2.039	(*111%*)	–
ThOC (mg/l C)	121.2	120.0	(*99%*)	122.5	(*101%*)	–
ThOD (mg/l O_2_)	306.7	319.7	(*104%*)	293.6	(*96%*)	–
BOD_5_ (mg/l O_2_)	179.9 ± 9.4	189.7 ± 8.0	(*105* ± *4%*)	173.2 ± 13.0	(*96* ± *7%*)	6.33 × 10^−3^
COD (mg/l O_2_)	282.0 ± 4.1	296.3 ± 2.7	(*105* ± *1%*)	266.0 ± 3.3	(*94* ± *1%*)	2.90 × 10^−4^
BOD_5_/COD ratio	0.61 ± 0.03	0.64 ± 0.03	(*105* ± *4%*)	0.58 ± 0.04	(*96* ± *7%*)	7.27 × 10^−7^
$\hat{I}$ (*μ*A/cm^2^)	96.5 ± 22.0	71.4 ± 19.8	(*74* ± *21%*)	109.9 ± 17.3	(*114* ± *18%*)	2.69 × 10^−2^
*Q*_*Cyc*_ (C/cm^2^)	53.0 ± 11.6	40.3 ± 11.1	(*76* ± *21%*)	66.7 ± 10.5	(*126* ± *20%*)	1.37 × 10^−2^
*C*_*E*_ (%)	34 ± 7%	25 ± 7%	(*73* ± *20%*)	43 ± 7%	(*128* ± *21%*)	8.87 × 10^−3^

† *p*-value from Welch's unequal variances *t*-test with a null hypothesis that the values from glucose and glutamic acid measurements have equal means. In all cases the null hypothesis was rejected at the 95% confidence level (*p*-value ¡ 0.05); indicating that the difference between glucose and glutamic acid measurements were statistically significant.

Each oxygen demand determination exhibited the same trend. ThOD values indicated that glucose medium had 4% higher carbonaceous demand than the equivalent GGA value, whereas glutamic acid medium demanded 4% less (Table 2). BOD_5_/COD ratios for glucose and glutamic acid of 0.640 and 0.584 respectively were statistically different, indicating glucose was more readily biodegraded as organic compounds are only partially consumed in the five-day BOD test.

The MFC response outputs ($\hat{I}$, *Q*_*Cyc*_ and *C*_*E*_) exhibited the opposite trend compared to the oxygen demand determinations (BOD_5_, COD and ThOD). Glucose values were approximately 25% less than corresponding GGA values and glutamic acid values were approximately 25% greater (Table 2; Figure S11a–c, ESI).

Less than half the substrate consumption generated electricity with coulombic efficiencies of only 25%, 43% and 33% with glucose, glutamic acid and GGA respectively (Table 2). Therefore, competitive substrate-consuming processes (e.g. fermentation or oxygen diffusion through the membrane leading to heterotrophic consumption) were occurring alongside the anodic biofilm. MFCs are not expected to achieve 100% *C*_*E*_ due to biomass production (85% *C*_*E*_ has been suggested as the likely maximum; Logan (2008)) and, with single-chamber MFCs especially, oxygen permeating the membrane is likely.

It is an important finding that despite MFC response correlating with oxygen demand for different concentrations of specific substrates (Figure S12, ESI), the same calibration cannot be applied for different compositions without re-calibration. Previously this has been attributed to biofilm acclimatisation and recommendations made to enrich MFCs with media similar to analyte composition (Kang et al., 2003). In the present work, biofilms were enriched with a 1:1 w/w mixture of glucose and glutamic acid, therefore the microbial community was selected capable of assimilating these substrates and generating electricity. This study demonstrates that different substrate responses cannot be solely attributed to acclimatisation (Table 2). These results demonstrate there are fundamental differences in oxidation rates in anaerobic MFCs leading to electricity generation compared with the conventional biological (aerobic microbial community) and chemical oxygen demand validations.

#### 3.3.3 Effect of real wastewater vs. synthetic wastewater at equal BOD

Two 28 L samples of real wastewater (WW1 and WW2) were collected from the influent to a treatment works. From each sample a 10 l sub-sample (suffixed ‘-A’) was taken for immediate analysis and the remainder (’-B’) was stored at 4 °C for 2 day as a reproducibility check. The pH, conductivity and MFC response was measured for each sub-sample. With the first sub-samples BOD_5_, COD, DOC and anion composition were also analysed (Table S3, ESI).

The BOD_5_ values of the real wastewater samples were 185.7 ± 8.7 mg/l O_2_ which was similar to the BOD_5_ value for the 300 mg/l GGA synthetic medium (179.9 ± 9.4 mg/l O_2_). BOD_5_/COD ratios and DOC values were lower for the real wastewater compared to the synthetic media (Table S3, ESI). COD was higher in real wastewater indicating the presence of less-biodegradable organic compounds. All wastewater samples were approximately pH 7 and fluoride and sulphate concentrations were higher. The WW1 bulk sample was apparently insufficiently homogenised as the non-adjusted conductivity of sub-sample WW1-A was very low (0.85 mS/cm) compared to WW1–B (3.95 mS/cm). The average conductivity of WW2 was 4.47 ± 0.01 mS/cm. In all cases, conductivity was adjusted to match synthetic media (increased to 8 mS/cm by adding approximately 2.6 ml/l of 2 mol/dm^3^ phosphate buffer (500 mg/l $PO_{4}^{3-}$)). Phosphate in the MFC tests was therefore closer to synthetic media.

The four sub-samples of real wastewater were tested with batch-mode MFCs C & D (Fig. 4a and S3, ESI), and the ‘DEF’ series of flow-mode MFCs (Fig. 4b and S6, ESI). In both cases, data from wastewater samples did not fit with the GGA calibration curves. Predicted BOD_5_ values were estimated from the measured current densities (Table S4, ESI). For the batch-mode sensors, in all cases except one, BOD_5_ was under-estimated by 3–69% (5–115 mg/l O_2_ BOD_5_). Whereas, flow-mode sensors under-estimated actual BOD_5_ values by 35–65%. The ‘B’ sub-samples both generated less electricity than their respective ‘A’ sub-samples indicating some degradation during low-temperature storage (23% less electricity generated with WW1 compared to 3% with WW2, potentially due to poor sample homogenisation).

An ordered response was observed in the predicted BOD_5_ values with the multi-stage flow-mode sensor (e.g. for sub-sample WW1-A (measured BOD_5_ = 186 ± 9 mg/l O_2_) the predictions from stages 1, 2 and 3 were 83 ± 126, 116 ± 65 and 185 ± 44 mg/l O_2_ respectively). As wastewater passed through the hydraulic array the prediction accuracy increased (third stage MFCs predicted values only 5% below the actual value of WW1 and 4% above WW2). However, the accuracy could be coincidental considering that the current density generated by the third stage MFCs was only 13.5 ± 4.5 and 12.6 ± 5.0 *μ*A/cm^2^ (8 and 7% of I_Max_) using WW1 and WW2 respectively. The response order observed in the hydraulically connected MFCs was similar to the behaviour observed with high concentrations of GGA medium which resulted in inhibition due to substrate excess (Section 3.1.2). Suggestive of competitive anaerobic processes occurring consuming substrate through non-electrogenic pathways, such as biomass production and non-electrogenic degradation of complex substrates to intermediates that were subsequently consumed electrogenically downstream in later stage MFCs. The pattern of electrical output observed with the flow-mode MFCs fed real wastewater could also indicate the presence of toxic compounds (Godain et al., 2020).

The MFC models in the present study calibrated with synthetic media could not accurately predict real wastewater BOD_5_ values. It is perhaps to be expected that the flow-mode sensors under-estimated more than the batch-mode sensors due to the lower incubation period of substrate in presence of the bioanode. Real wastewater contains polymeric substrates that can be hydrolysed and aerobically degraded within the time-scales of the BOD_5_ test, yet the hydraulic retention time in the MFC sensor array is substantially lower (40 min).

Anaerobic oxidation of GGA-based medium in MFCs did not resemble similar oxidation rates observed with real wastewater in the aerobic BOD_5_ test. In the standard BOD_5_ test GGA is used as the calibrant and oxidation rates are assumed to be similar (although BOD_5_/COD ratios indicate this is inaccurate; Table S3, ESI). Whilst the MFC sensors had excellent sensing characteristics for fixed medium compositions under well defined conditions, it is demonstrated here that such sensors should be enriched and calibrated with real wastewaters for accurate BOD_5_ prediction (existing synthetic wastewater proxies are not suitable).

As wastewater is a complex mixture of many different compounds it is unlikely that the electrical output would be dominated by any single compound. Therefore, natural fluctuations are unlikely to lead to dramatic changes in sensor output (such as was observed switching from 100% glucose to 100% glutamic acid-based media). The complex composition of wastewater is the reason that oxygen demand (rather than compound concentration) is assessed conventionally and evidently the rates at which this is anaerobically converted to electrogenic substrates differs. Nonetheless, MFC sensor measurements complement measures of composite biodegradable organic matter which could revolutionise water quality monitoring, even if calibration models need to take into account wastewater composition to accurately predict BOD_5_ values.

### 3.4 Microbial communities on electrodes

Microbial communities from MFC sensor anodes were characterised by sequencing 16S rRNA genes. Communities were analysed from batch-mode MFCs A and B (after 848 days operation) and non-polarised OCP electrodes A, B and C (after 36 days at open circuit).

Communities from the electrodes were compared using principal co-ordinate analysis of unweighted Unifrac distances. Communities clustered on the basis of electrode polarisation and the first and second principal co-ordinates accounted for 39% of the variance between batch-mode anode and OCP electrode communities (Figure S13, ESI). MFC B achieved greater electrochemical performance than MFC A (Figure S8, ESI), positioned further from the OCP samples (Figure S13, ESI) and had a higher relative abundance of *Geobacter* spp. (Figure S14, ESI), a genus that includes well known electrogens (Rotaru et al., 2015).

Bacteria from the genus *Geobacter* (26% relative abundance) and *Porphyromonadaceae* family (39%) were selectively enriched by 2-3 orders of magnitude at levels of 10^8^–10^9^ cells per cm^2^. Unclassified *Cryomorphaceae* (4%) were exclusively enriched on polarised electrodes.

Bacteria from the genus *Anaeromusa* (associated with glutamate fermentation (Ouattara et al., 1992)) and members of *Enterobacteriaceae* were present in much higher relative abundance on OCP electrodes where anodic electron accepting reactions could not occur (respectively 30% and 39% at OCP compared to 3% and ¡1% on bioanodes).

Microbial fermentation and methanogenesis are key processes leading to coulombic losses which do not occur aerobically (Torres et al., 2007; Kim et al., 2011). Several genera were common to both polarised and non-polarised electrodes but at higher relative abundance (2–8%) at OCP and likely performed non-electrogenic, auxiliary functions. Functions including glucose fermentation (e.g. *Tolumonas* spp.; Tindall, 1996 and *Dysgonomonas* spp.; Kodama et al., 2012) and glutamate fermentation (*Desulfovibrio* spp.; Stams and Hansen, 1984). The only methanogenic archeon detected, *Methanobrevibacter arboriphilus*, was present at only 0.06% relative abundance on bioanodes indicating that methanogenesis was probably minimal.

Similarly, electrode microbial communities from the ‘ABC’ series of flow-mode MFCs and non-anode associated biomass sludge were characterised previously in Spurr et al. (2018). In brief, the electrogenic community of flow-mode bioanodes included *Geobacter* spp. (24–50%) and unclassified *Porphyromonadaceae* (5–12% in MFC stages 1–3). The anodic bacterial community also contained amino acid fermenters (*Anaeromusa* spp., *Desulfovibrio* spp. and members of *Comamonadaceae*) and sugar fermenters (*Tolumonas* spp., *Dysgonomonas* spp., *Lactococcus* spp. and members of *Enterobacteriaceae*). Notably, the community composition of the biomass sludge rapidly changed at high GGA concentrations (2000 mg/l) with *Lactococcus* spp. (likely glucose fermenters) increasing from 48% to 88–95% relative abundance.

Less amino acid fermenting bacteria than sugar fermenting bacteria were found on batch-mode electrodes. Therefore, potentially there was less glutamic acid consumption by competitive processes; thus greater coulombic efficiency was achieved and MFCs reached substrate saturation at lower concentrations. This supports the finding that MFCs could respond to higher concentrations of glucose than glutamic acid (as more is lost non-electrogenically).

## 4 Conclusions

It has been shown that for both batch- and flow-mode MFCs it is possible to reliably utilise their response for BOD sensing without re-calibration over years of operation and across independent replicates. However, this relies upon operating and environmental conditions remaining fixed, including the analyte (medium composition), external resistance and other operating conditions (i.e. temperature, flow rate). This has important implications for long term operational stability and re-calibration requirements of commercial MFC sensing prospects.

The effect that increasing R_Ext_ above the internal resistance has on decreasing calibration range has been established. Furthermore, the effect of changing the carbon source has been comprehensively assessed in synthetic and real wastewaters. It has been shown that biofilm acclimatisation is not responsible for differences between aerobic oxygen demand determinations and anaerobic MFC responses and these are likely attributable to competitive processes such as fermentation. The necessity to calibrate with a medium similar in composition to the analyte was demonstrated, as existing synthetic calibrants were found to under-estimate BOD_5_ values of real wastewaters. Further work could focus on expanding the complexity and range of substrates tested beyond simple, readily-consumed compounds and with realistic/unamended conductivities to enhance understanding of bioanode oxidation in comparison to conventional oxygen demand tests. This study indicates the potential of MFC sensors for real-time organic load monitoring and process control in treatment processes.

## CRediT authorship contribution statement

**Martin WA. Spurr:** Conceptualization, Validation, Methodology, Formal analysis, Data curation, Investigation, Writing – original draft, Visualization, Project administration. **Eileen H. Yu:** Validation, Writing – review & editing, Supervision, Funding acquisition. **Keith Scott:** Validation, Writing – review & editing, Supervision, Funding acquisition. **Ian M. Head:** Conceptualization, Validation, Methodology, Writing – review & editing, Supervision, Project administration, Funding acquisition.

## Declaration of competing interest

The authors declare that they have no known competing financial interests or personal relationships that could have appeared to influence the work reported in this paper.
