# Supplementary material for: No re-calibration required? Stability of a bioelectrochemical sensor for biodegradable organic matter over 800 days
Source: Biosens Bioelectron. 2021 Oct 15;190:None. doi: 10.1016/j.bios.2021.113392 (PMC8316843; doi:10.1016/j.bios.2021.113392)
Supplement: Multimedia component 1 [file mmc1.pdf]

Supplementary Material

Section S1: Polarisation curves method

Cells were put into open circuit by disconnecting the external resistor and time was allowed for a steady-state open circuit potential to be reached (up to 3 hours or when voltage change was less than +10 µV/s) before the cell and anode potentials were monitored. To each cell an external resistor was connected in the sequence; 52 300, 12 700, 5100, 1820, 953, 503, 305, 200, 100, 44 and 10.6 Ω. Approximately 20 minutes was allowed between each resistor change in order for the voltages to stabilise and be recorded. Following the final measurement, the original resistor used for MFC operation was connected and normal operation was resumed.

Section S2: Community analysis details:

PCR was performed for one cycle at 95 °C (04:00 min), 30 cycles at 95 °C (01:00), 55 °C (00:45), 72 °C (01:00) and a final cycle at 72 °C (10:00). A pooled amplicon library was created by combining 26 pmol/dm^3 amplified 16S rRNA gene fragments from each sample and clonal amplification on microbeads was achieved by emulsion PCR. Microbeads were loaded onto an Ion 316 microchip.

Operational taxonomic units (OTUs) were picked using the uclust method (Edgar, 2010) with reference to the Greengenes database (at 97% identity), followed by denovo picking for unmatched sequences. Taxonomic assignment was performed on representative sequences using the RDP classification algorithm (Wang et al., 2007). Sequences were aligned to a PyNAST template and filtered. The ribosomal RNA operon database (rrnDB) was used to adjust 16S rRNA gene abundance data with respect to 16S rRNA gene copy number in different organisms, to more accurately reflect the relative abundance of the population (>80% confidence (Stoddard et al., 2015)). Unifrac distance matrices based on community composition were calculated and abundance-weighted for comparative analysis of the microbial communities.

Accession numbers for sequences in this dataset available from GenBank Nucleotide database:

- MF979803.1 Uncultured Methanobrevibacter sp. clone OTU547463 16S ribosomal RNA gene, partial sequence
- MF979802.1 Uncultured Geobacter sp. clone OTU19 16S ribosomal RNA gene, partial sequence
- MF979801.1 Uncultured Citrobacter sp. clone OTU814442 16S ribosomal RNA gene, partial sequence
- MF979800.1 Uncultured Cryomorphaceae bacterium clone OTU809916 16S ribosomal RNA gene, partial sequence
- MF979799.1 Uncultured Enterobacteriaceae bacterium clone OTU691423 16S ribosomal RNA gene, partial sequence
- MF979798.1 Uncultured Enterobacteriaceae bacterium clone OTU676211 16S ribosomal RNA gene, partial sequence
- MF979797.1 Uncultured Anaeromusa sp. clone OTU588869 16S ribosomal RNA gene, partial sequence
- MF979796.1 Uncultured Desulfovibrio sp. clone OTU513307 16S ribosomal RNA gene, partial sequence
- MF979795.1 Uncultured Porphyromonadaceae bacterium clone OTU4466138 16S ribosomal RNA gene, partial sequence

- MF979794.1 Uncultured Tolumonas sp. clone OTU4439030 16S ribosomal RNA gene, partial sequence
- MF979793.1 Uncultured Enterobacteriaceae bacterium clone OTU4418165 16S ribosomal RNA gene, partial sequence
- MF979792.1 Uncultured Trabulsiella sp. clone OTU4406193 16S ribosomal RNA gene, partial sequence
- MF979791.1 Uncultured Enterobacteriaceae bacterium clone OTU4328189 16S ribosomal RNA gene, partial sequence
- MF979790.1 Uncultured Comamonadaceae bacterium clone OTU4297171 16S ribosomal RNA gene, partial sequence
- MF979789.1 Uncultured Comamonadaceae bacterium clone OTU366977 16S ribosomal RNA gene, partial sequence
- MF979788.1 Uncultured Dysgonomonas sp. clone OTU2634854 16S ribosomal RNA gene, partial sequence
- MF979787.1 Uncultured Enterobacteriaceae bacterium clone OTU228556 16S ribosomal RNA gene, partial sequence
- MF979786.1 Uncultured Geobacter sp. clone OTU1725261 16S ribosomal RNA gene, partial sequence
- MF979785.1 Uncultured Lactococcus sp. clone OTU1130824 16S ribosomal RNA gene, partial sequence
- MF979784.1 Uncultured Geobacter sp. clone OTU1102952 16S ribosomal RNA gene, partial sequence

Edgar, R.C., 2010. Search and clustering orders of magnitude faster than BLAST. Bioinformatics 26, 2460–2461. https://doi.org/10.1093/bioinformatics/btq461

Stoddard, S.F., Smith, B.J., Hein, R., Roller, B.R.K., Schmidt, T.M., 2015. rrnDB: Improved tools for interpreting rRNA gene abundance in bacteria and archaea and a new foundation for future development. Nucleic Acids Res. 43, D593–D598. https://doi.org/10.1093/nar/gku1201

Wang, Q., Garrity, G.M., Tiedje, J.M., Cole, J.R., 2007. Naive Bayesian classifier for rapid assignment of rRNA sequences into the new bacterial taxonomy. Appl. Environ. Microbiol. 73, 5261–5267. https://doi.org/10.1128/AEM.00062-07

Table S 1: Model parameters determined from calibrations over time performed using batch-mode cells A, B, C and D with R_{Ext} = 43.2 Ω. Parameter values are given with precision ± SD.

Calibration ID (MFC series)	Time since inoculation (days)	Linear BOD_5 range[a] (mg/l O_2)	Linear model $y = mx + c$				Hill model $v = \frac{v_{Max}[S]^h}{K_M^h + [S]^h}$			
			No. cycles[b]	*m* (% / (mg/l O_2))	*c* (mg/l O_2)	R^2	v_{Max} (%)	K_M (mg/l O_2)	*h*	R^2
1 (A&B)	136	30-180	5	0.434 ± 0.026	1.43 ± 2.67	0.989	133 ± 73	151.0 ± 146.0	1.37 ± 0.84	0.980
2 (A&B)	219	16-150	7	0.485 ± 0.068	5.94 ± 5.95	0.911	105 ± 19	82.9 ± 46.0	1.60 ± 1.29	0.942
3 (A&B)	328	15-240	16	0.397 ± 0.017	7.28 ± 2.55	0.975	112 ± 20	103.6 ± 31.9	1.77 ± 0.88	0.974
4 (A&B)	730	15-240	16	0.345 ± 0.036	9.69 ± 5.41	0.866	106 ± 28	107.9 ± 50.6	1.67 ± 1.40	0.907
5 (A&B)	792	60-300	7	0.400 ± 0.028	0.31 ± 5.11	0.986	104 ± 14	115.5 ± 39.6	2.61 ± 1.57	0.868
6 (C&D)	56	15-240	13	0.442 ± 0.044	3.34 ± 6.38	0.901	109 ± 38	103.3 ± 58.0	2.08 ± 2.83	0.879
Combined	-	15-240	64	0.391 ± 0.016	7.05 ± 2.24	0.911	106 ± 22	99.7 ± 44.0	1.92 ± 1.55	0.918

[a] Estimated BOD_5 (from GGA concentration). [b] Number of medium replacement cycles (from which peak current densities were determined) included in the linear model.

Table S 2: Average response time to reach 95% peak current density at different R_{Ext} using calibration data (for medium cycles < 750 mg/l O_2 BOD_5 for response times and cycles which had run to completion for $\%_{CE3}$) from batch-mode cells A, B, C & D.

R_{Ext}	№ cycles [a] (< 750 mg/l O_2)	95% response time (+SD)	Coulombic efficiency (+SD)
43.2 Ω	86	2.3 +1.6 hours	37 +17 %
305 Ω	22	1.9 +1.4 hours	25 +15 %
953 Ω	15	1.5 +0.8 hours	21 +14 %
5100 Ω	14	1.4 +1.4 hours	9 +6 %

[a] Number of cycles included in the response time calculation.

Table S 3: Comparison of water quality parameters for a 300 mg/l GGA synthetic wastewater medium and two samples of real raw influent wastewater (WW1 and WW2) of which two sub-samples were taken (A and B). Water quality measurements were taken using the 'A' sub-sample on the day of wastewater collection.

Parameter	300 mg/l GGA synthetic WW	WW1 'A'	WW1 'B'	WW2 'A'	WW2 'B'
Volume prepared (l)	5.6	11.0	16.3	10.0	18.0
pH	6.96	7.04	6.73	7.30	7.26
Conductivity (mS/cm)	-	0.85	3.95	4.46	4.48
Adjusted conductivity (mS/cm) [a]	7.40	8.49	8.26	8.41	8.18
BOD_5 (mg/l O_2)	179.9 ± 9.4	185.7 ± 8.7		168.1 ± 6.4	
COD (mg/l O_2)	282.0 ± 4.1	450.5 ± 13.4		544 ± 2	
BOD5/COD ratio	0.607 ± 0.031	0.413 ± 0.032		0.309 ± 0.026	
DOC (mg/l C)	113.4 ± 1.2	82.5 ± 3.3		97.9 ± 3.8	
IC (mg/l C)	ND	53.8 ± 0.4		63.3 ± 1.3	
Fluoride (mg/l)	ND	6.5 ± 2.1		6.7 ± 2.2	
Chloride (mg/l)	63.7 ± 2.9	65.3 ± 0.5		193.8 ± 150.3	
Phosphate (mg/l)	1860.2 ± 210.3	24.5 ± 1.9		34.4 ± 14.9	
Sulphate (mg/l)	11.2 ± 1.8	65.1 ± 0.6		110.6 ± 42.5	

[a] Wastewater samples were adjusted by titrating an appropriate amount of 2 mol/dm3 phosphate buffer to bring conductivity to approximately 8 mS/cm. [b] ND = Not detected.

Table S 4: Evaluation of predicted BOD_5 values using the G, GA and GGA calibrated linear and Hill models from the batch-mode MFCs for four samples of real wastewater. Mean percentages are given in relation to the actual BOD_5 value measured by the standardised test.

Sample	$\hat{I}$ ($\mu A/cm^2$) [a,c]	BOD_5 (mg/l O_2) [c,d]	Model	Predicted BOD5 (mg/l O_2) by… GGA model: Value [c]	GGA model: % of BOD_5 [b,c]	G model: Value [c]	G model: % of BOD_5 [b,c]	GA model: Value [c]	GA model: % of BOD_5 [b,c]
300 mg/l GGA	160.6 ± 16.5	180 ± 9	Linear	191 ± 39	(106 ± 22%)	263 ± 30	(146 ± 17%)	152 ± 25	(84 ± 14%)
			Hill	177 ± 34	(99 ± 19%)	308±124	(171 ± 69%)	146 ± 44	(81 ± 24%)
WW1-A	104.1 ± 2.7	186 ± 9	Linear	138 ± 37	(74 ± 20%)	190 ± 27	(102 ± 14%)	105 ± 23	(56 ± 13%)
			Hill	119 ± 15	(64 ± 8%)	181 ± 45	(97 ± 24%)	65 ± 16	(47 ± 11%)
WW1-B	79.7 ± 0.1	-	Linear	103 ± 36	(56 ± 20%)	142 ± 26	(77 ± 14%)	74 ± 24	(40 ± 13%)
			Hill	95 ± 12	(51 ± 6%)	132 ± 28	(71 ± 15%)	65 ± 16	(35 ± 9%)
WW2-A	66.3 ± 8.0	168 ± 6	Linear	84 ± 37	(50 ± 22%)	115 ± 26	(69 ± 15%)	57 ± 25	(34 ± 15%)
			Hill	83 ± 12	(49 ± 7%)	110 ± 24	(65 ± 14%)	55 ± 16	(32 ± 9%)
WW2-B	64.0 ± 7.1	-	Linear	80 ± 37	(48 ± 22%)	111 ± 26	(66 ± 15%)	54 ± 25	(32 ± 15%)
			Hill	81 ± 12	(48 ± 7%)	106 ± 24	(63 ± 14%)	53 ± 16	(31 ± 9%)

[a] $\hat{I}$ = Average peak current density.
[b] Mean percentage is calculated from the fitted values as a percentage of the actual BOD_5.
[c] Values are reported "± ½ Range" from the 95% prediction interval (models) or measured replicates.
[d] The BOD_5 test was not repeated between 'A'-suffixed samples (immediate MFC analysis) and 'B'-suffixed samples (stored at 4 °C for 2 days prior to MFC analysis).

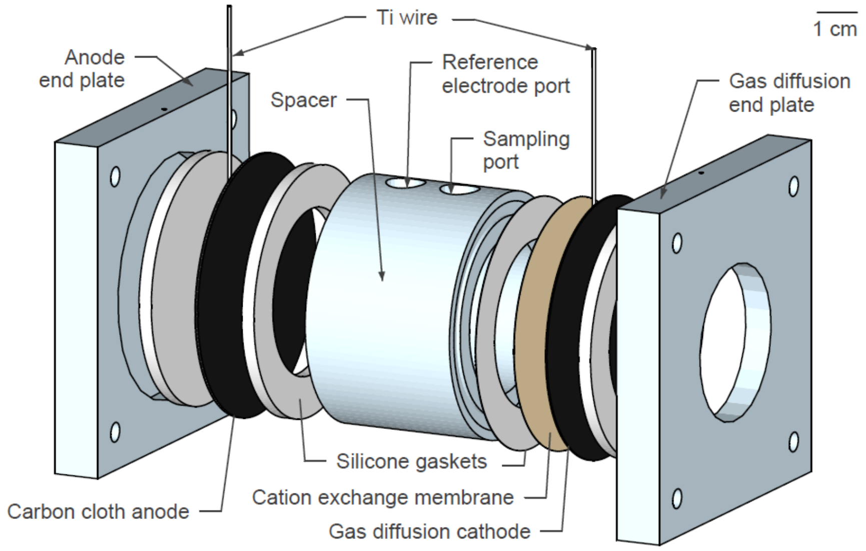

Figure S 1: Diagram of 50 ml batch-mode MFC.

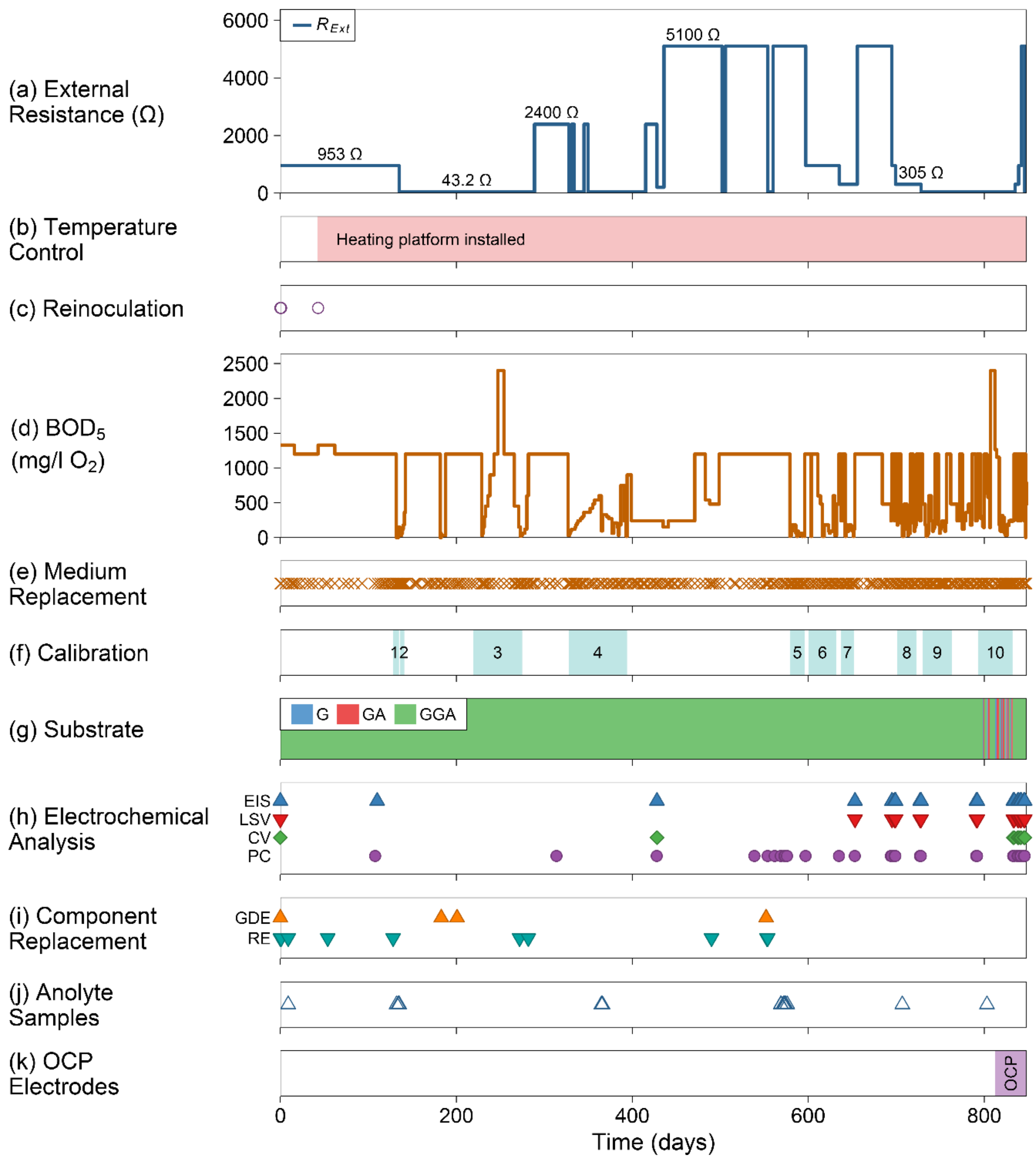

Figure S 2: Timeline of events summarising the operation of batch-mode MFCs (A and B) and OCP electrodes including (a) external resistance used for MFC operation, (b) temperature control installation, (c) reinoculation events, (d) known BOD_5 medium concentrations, (e) medium replacement events, (f) labelled calibration bands representing range of included batch cycles, (g) medium substrate type, (h) electrochemical analysis, (i) component replacement, (j) analyte sample events and (k) band representing length of OCP electrode operation.

G = Glucose; GA = Glutamic acid; GGA = Glucose-Glutamic acid; EIS = Electrochemical Impedance Spectroscopy; LSV = Linear Sweep Voltammetry; CV = Cyclic Voltammetry; PC = Polarisation Curve; GDE = Gas Diffusion Electrode; RE = Reference Electrode.

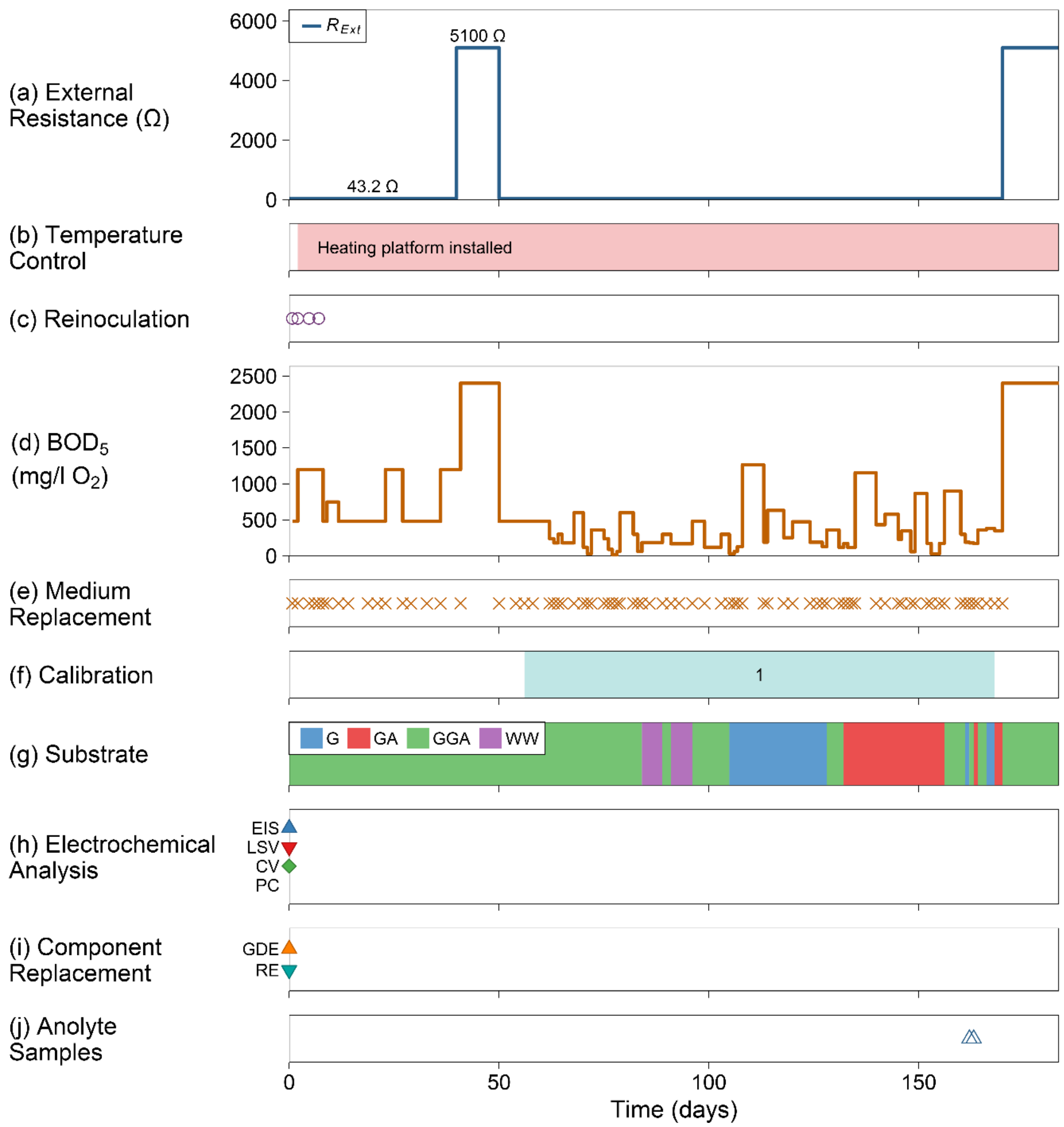

Figure S 3: Timeline of events summarising the operation of batch-mode MFCs (C and D) including (a) external resistance used for MFC operation, (b) temperature control installation, (c) reinoculation events, (d) known BOD_5 medium concentrations, (e) medium replacement events, (f) labelled calibration bands representing range of included batch cycles, (g) medium substrate type, (h) electrochemical analysis, (i) component replacement and (j) anolyte sample events.

G = Glucose; GA = Glutamic acid; GGA = Glucose-Glutamic acid; WW = Wastewater; EIS = Electrochemical Impedance Spectroscopy; LSV = Linear Sweep Voltammetry; CV = Cyclic Voltammetry; PC = Polarisation Curve; GDE = Gas Diffusion Electrode; RE = Reference Electrode.

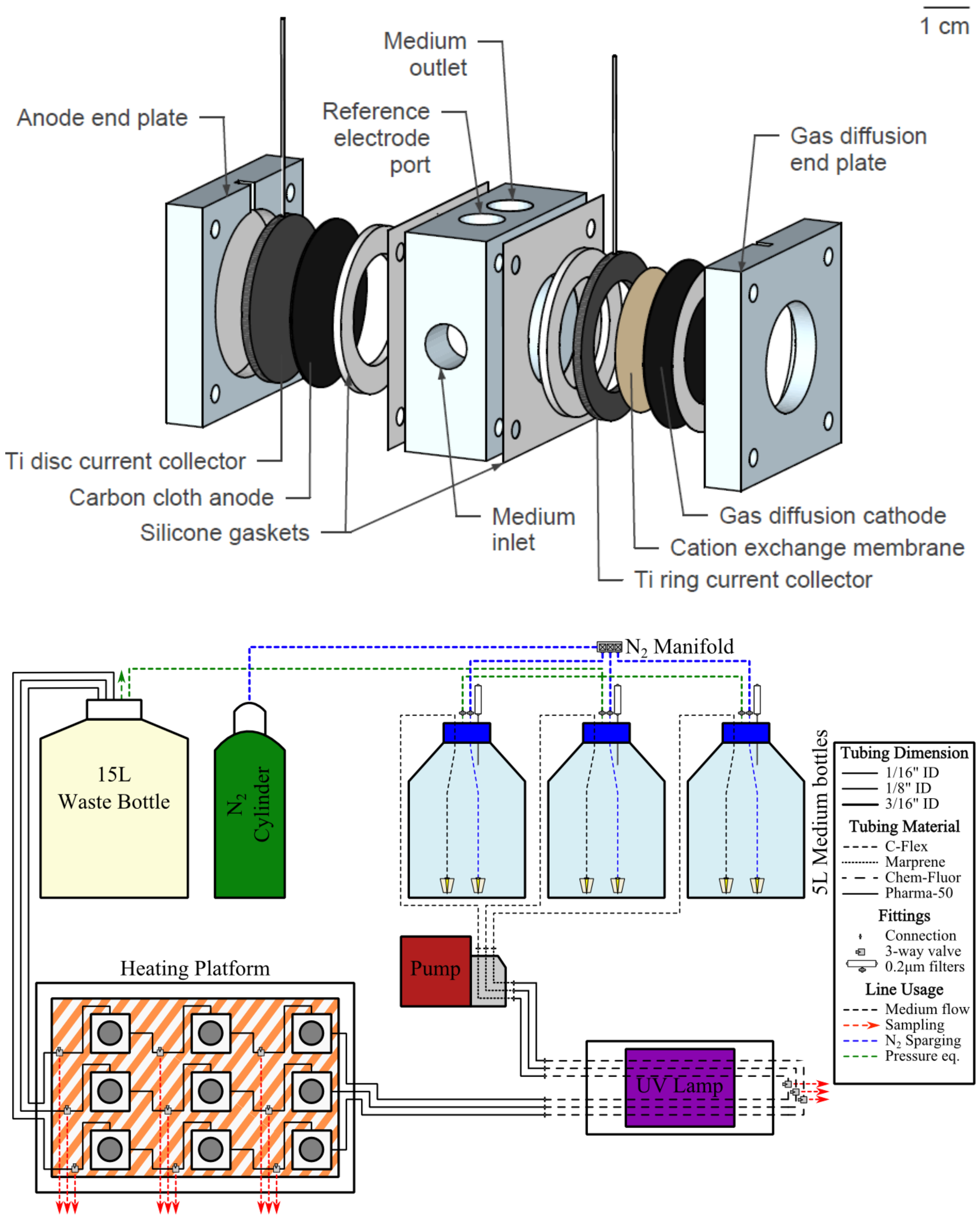

Figure S 4: Diagrams of (A) 10 ml flow-mode MFC and (B) Triplicate three-stage MFC cascade setup.

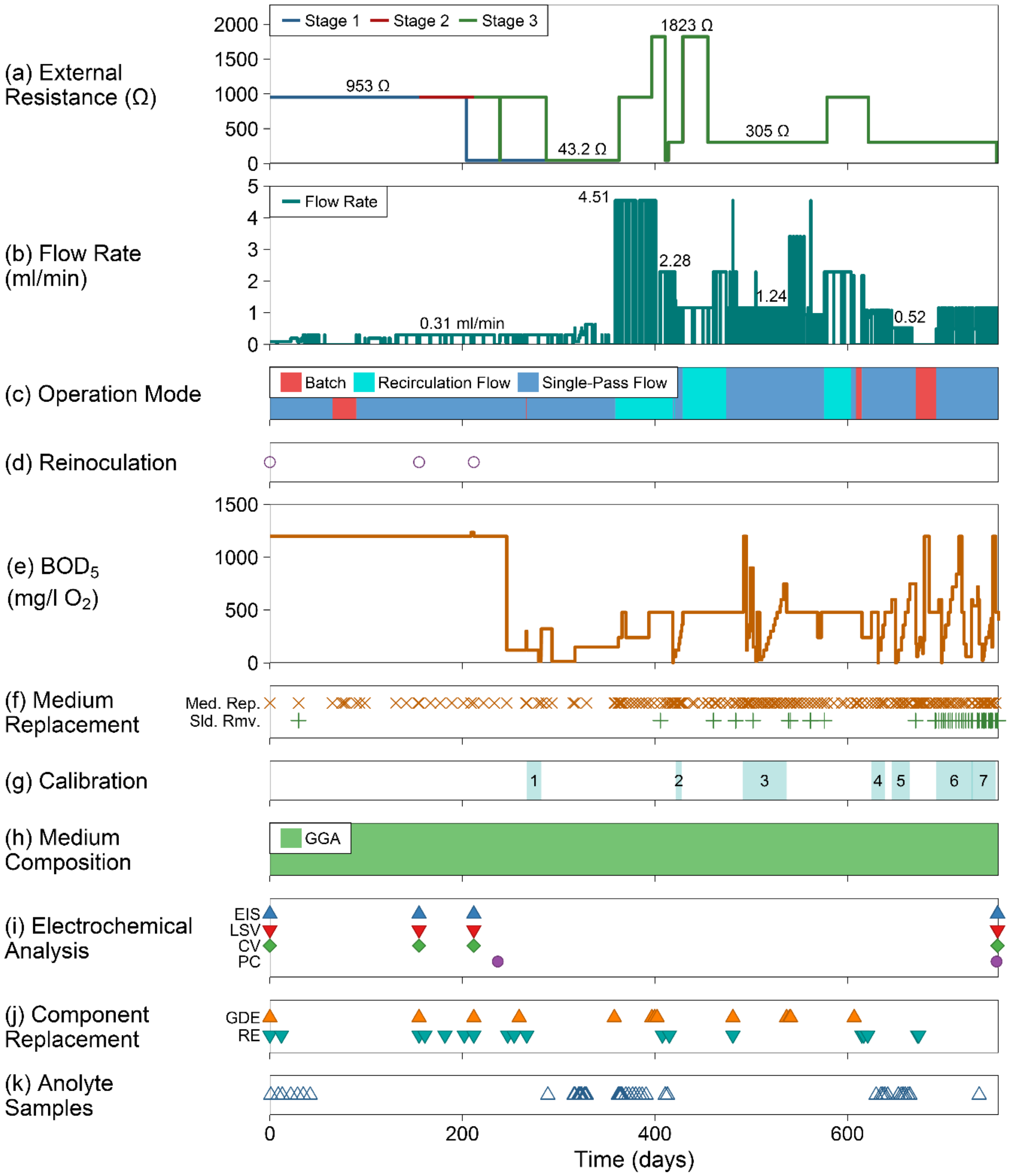

Figure S 5: Timeline of events summarising the operation of flow-mode MFCs (1A, 1B, 1C, 2A, 2B, 2C, 3A, 3B and 3C) including (a) external resistance used for MFC operation, (b) medium flow rate, (c) mode of operation, (d) reinoculation events, (e) BOD_5 (estimated from known GGA concentrations), (f) medium replacement events, (g) labelled calibration bands representing range of included medium replacements, (h) medium composition, (i) electrochemical analysis, (j) component replacement, (k) anolyte sample events.

Med. Rep. = Medium Replacement, Sld. Rmv. = Sludge Removal, GGA = Glucose-Glutamic acid; EIS = Electrochemical Impedance Spectroscopy; LSV = Linear Sweep Voltammetry; CV = Cyclic Voltammetry; PC = Polarisation Curve; GDE = Gas Diffusion Electrode; RE = Reference Electrode.

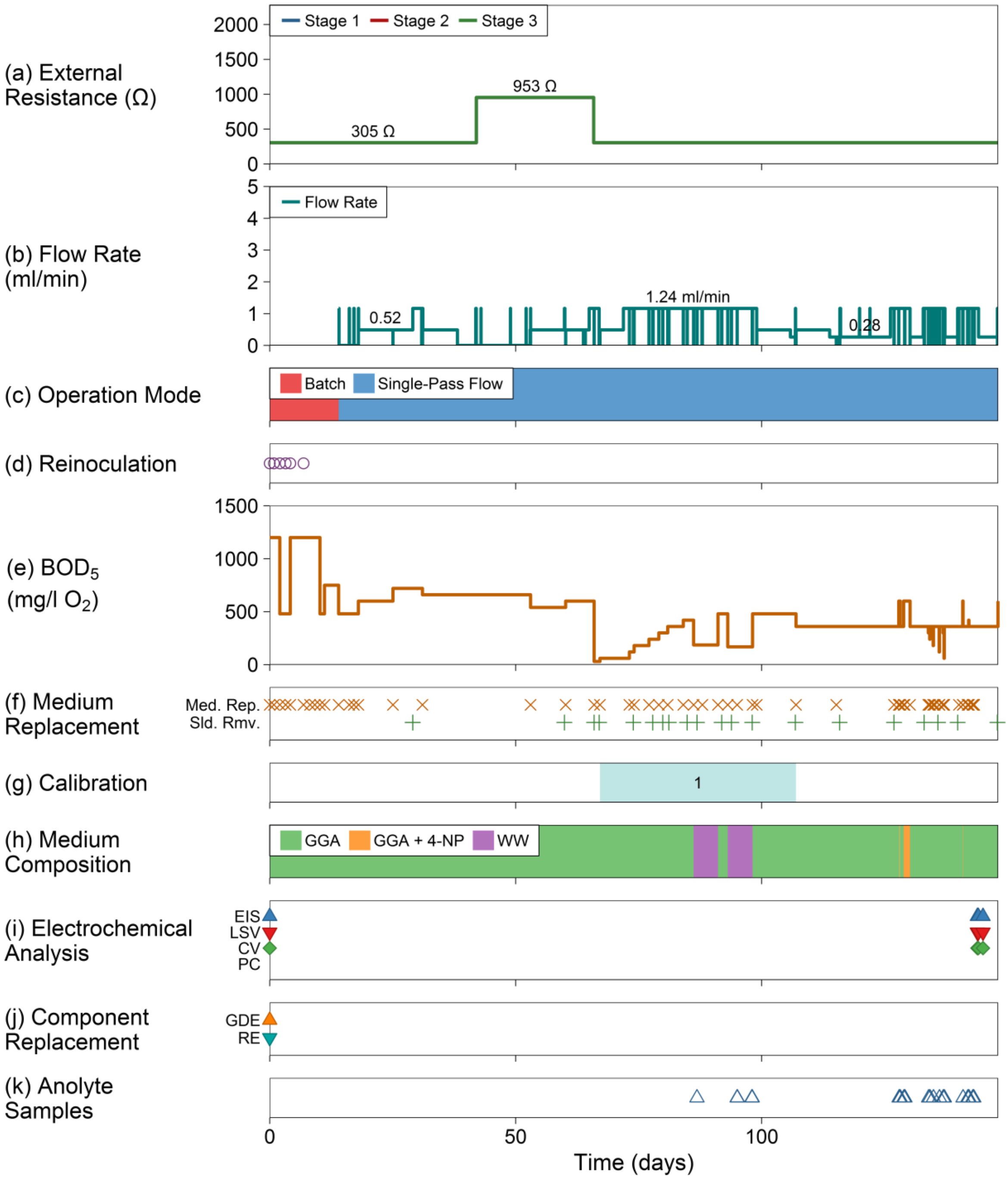

Figure S 6: Timeline of events summarising the operation of flow-mode MFCs (1D, 1E, 1F, 2D, 2E, 2F, 3D, 3E and 3F) including (a) external resistance used for MFC operation, (b) medium flow rate, (c) mode of operation, (d) reinoculation events, (e) BOD_5 (estimated from known GGA concentrations), (f) medium replacement events, (g) labelled calibration bands representing range of included medium replacements, (h) medium composition, (i) electrochemical analysis, (j) component replacement, (k) analyte sample events.

Med. Rep. = Medium Replacement, Sld. Rmv. = Sludge Removal, GGA = Glucose-Glutamic acid; 4-NP = 4-Nitrophenol; WW = Wastewater; EIS = Electrochemical Impedance Spectroscopy; LSV = Linear Sweep Voltammetry; CV = Cyclic Voltammetry; PC = Polarisation Curve; GDE = Gas Diffusion Electrode; RE = Reference Electrode.

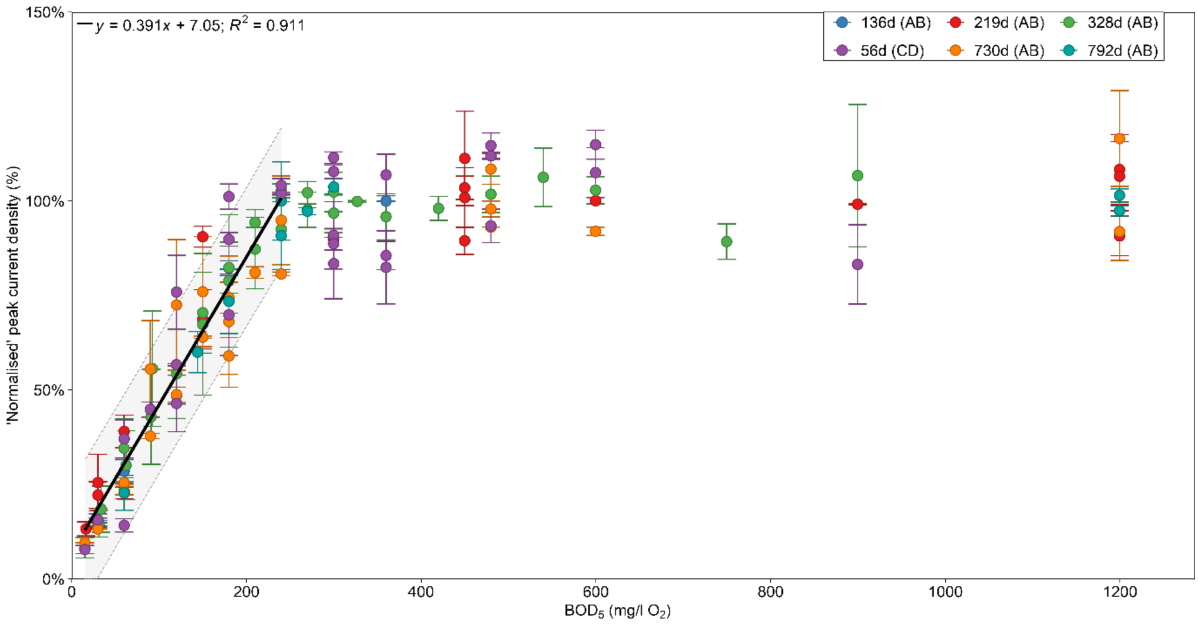

Figure S 7: Average peak current density calibration data (normalised by maximum current density) against BOD_5 (estimated from GGA concentration) obtained during calibrations at different time points during operation of batch-mode MFCs A, B, C and D with R_{Ext} = 43.2 Ω fitted with a linear model. The legend states the calibration starting day and MFC series used. The shaded band represents the 95% prediction interval from the linear model and error bars are the range of values from duplicate cells.

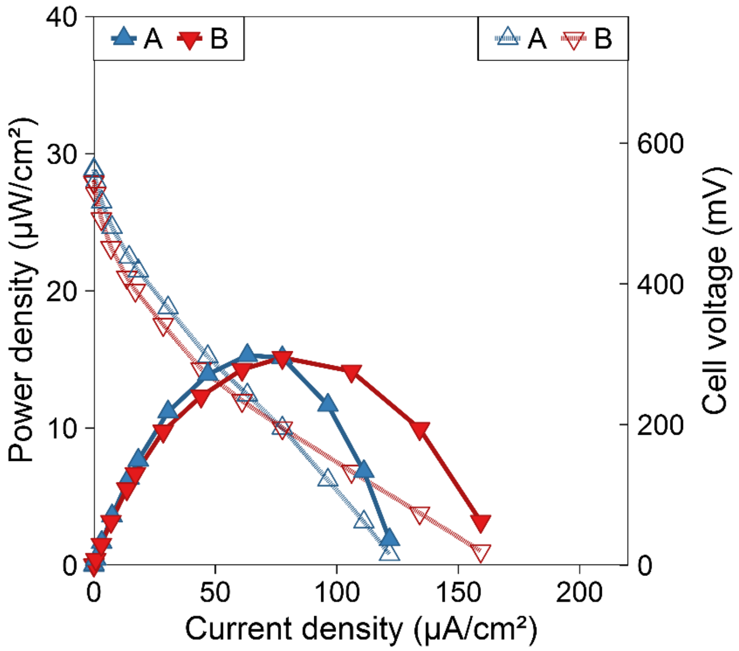

Figure S 8: Polarisation and power density curves recorded from batch-mode MFCs A & B after 792 days of operation. In both MFCs peak power was achieved over a R_{Ext} of 305 ohms.

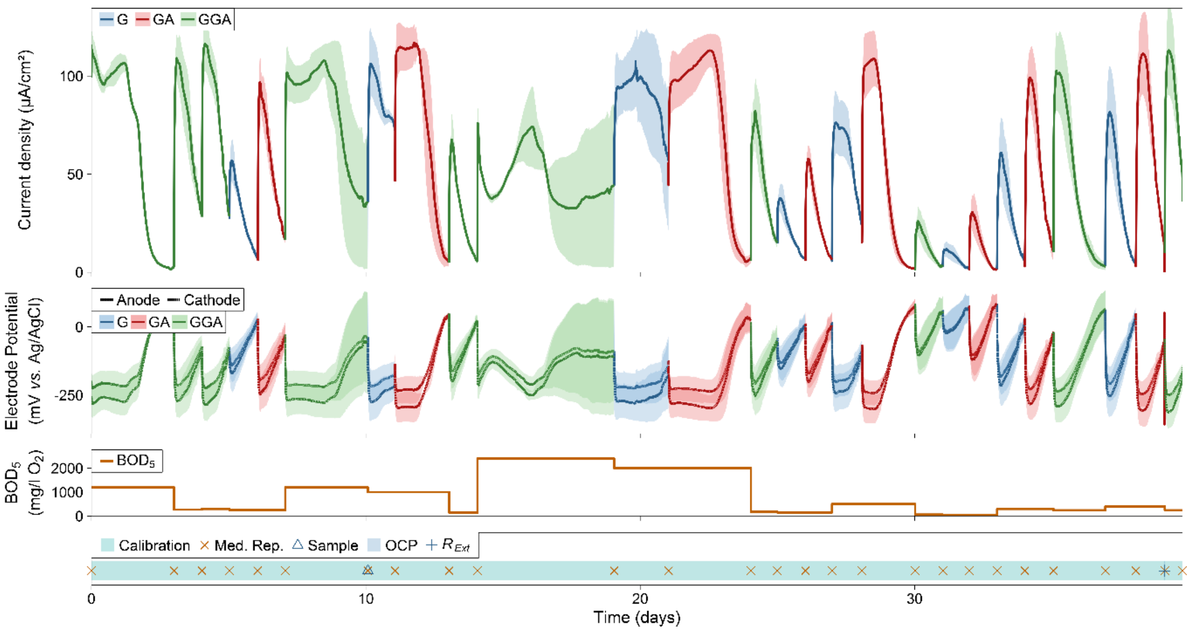

Figure S 9: The average response of batch-mode MFCs A and B current density, anode and cathode potential to changes in BOD_5 (estimated from substrate concentration) with each medium replacement during Calibration 10 in Figure S 2. Coloured lines represent medium composition of glucose (G), glutamic acid (GA) and GGA.

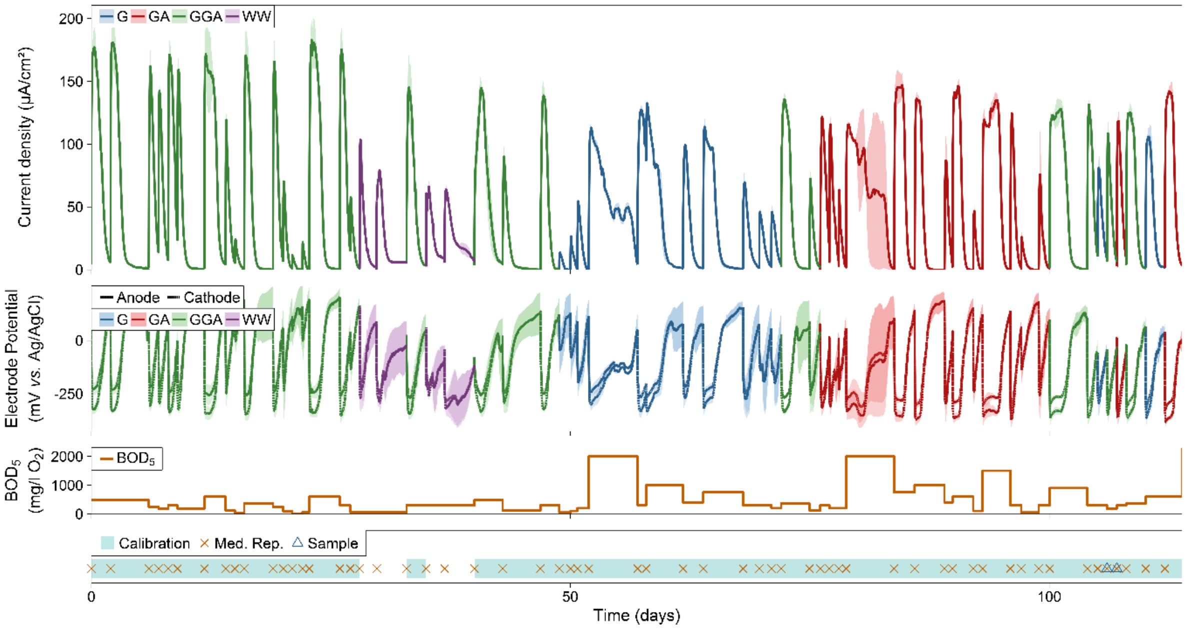

Figure S 10: The average response of batch-mode MFCs C and D current density, anode and cathode potential to changes in BOD_5 (estimated from substrate concentration) with each medium replacement during Calibration 1 in Figure S 3. Coloured lines represent medium composition of glucose (G), glutamic acid (GA), GGA and wastewater (WW).

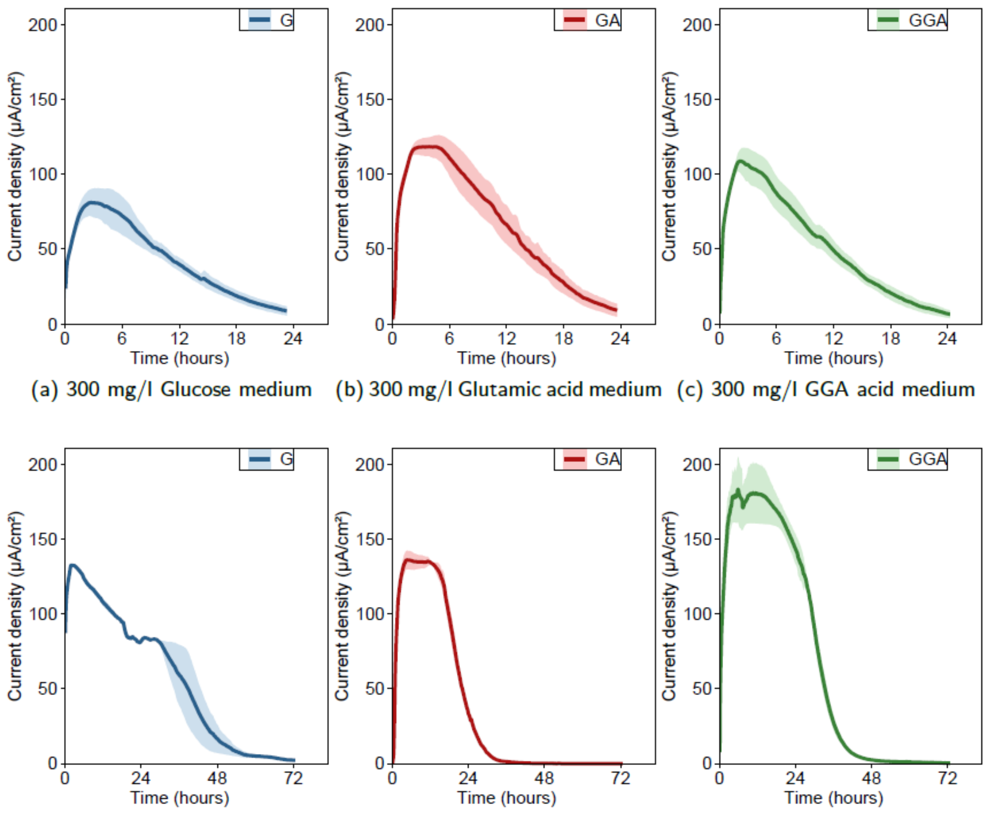

(a) 300 mg/l Glucose medium (b) 300 mg/l Glutamic acid medium (c) 300 mg/l GGA acid medium

(d) 1000 mg/l Glucose medium (e) 1000 mg/l Glutamic acid medium (f) 1000 mg/l GGA medium

Figure S 11: Average current density response for batch cycles in which 300 mg/l (a) glucose, (b) glutamic acid and (c) GGA and 1000 mg/l (d) glucose, (e) glutamic acid and (f) GGA was fed to batch-mode MFCs C & D. 300 mg/l cycles were recorded between 161–164 days of operation where cathode degradation was negligible and thus current density values in (a–c) can be compared, whereas 1000 mg/l cycles were recorded on days 113, 141 and 67 of operation respectively during which time a fall in cathode potential was observed; therefore current density magnitudes in (d–f) should not be compared. Shaded bands are the S5range between cell C & D.

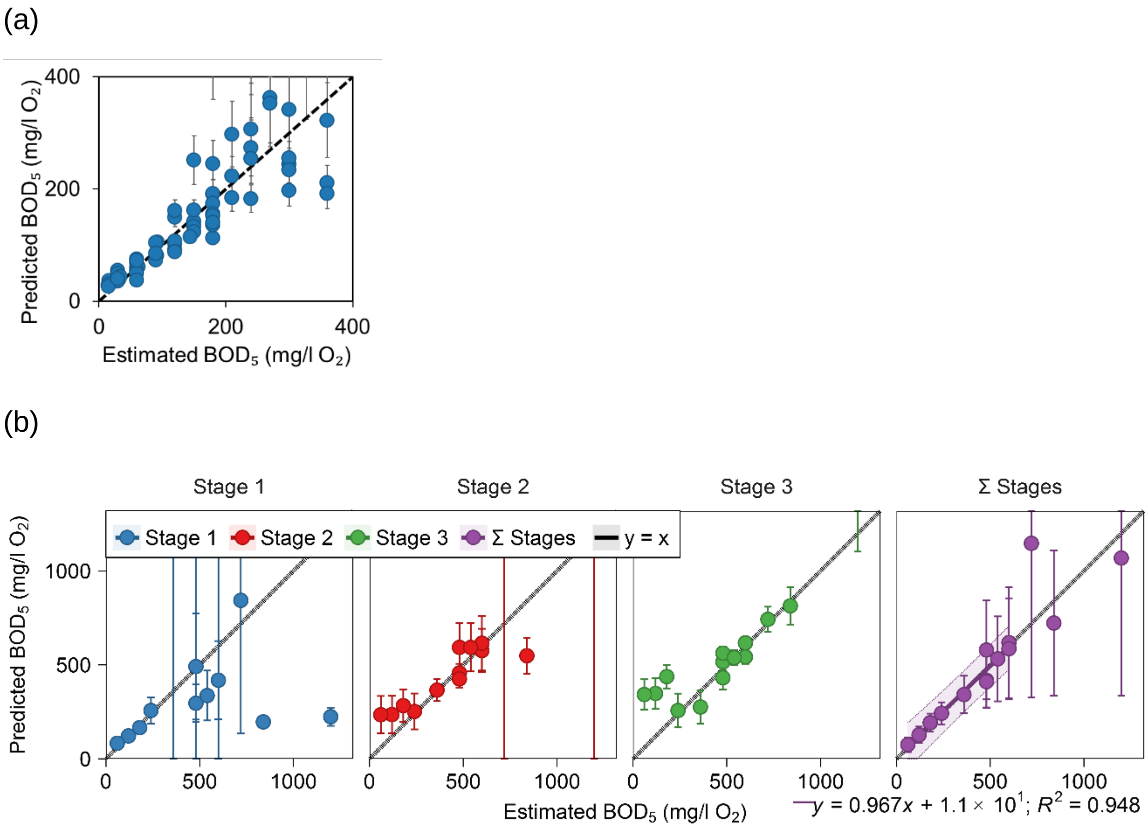

Figure S 12: Predicted BOD_5 plotted against estimated BOD_5 (from GGA concentration) for values predicted by the Hill calibration models using current densities obtained during calibrations of (a) batch-mode MFCs A, B, C & D and (b) the flow-mode 'ABC' MFC stages. y = x is shown as the 'ideal' prediction. Outliers with a Predicted BOD_5 above 450 and 1000 mg/l O_2 (batch and flow respectively) were removed from the Hill plot as the error bars were outside the limits of the model.

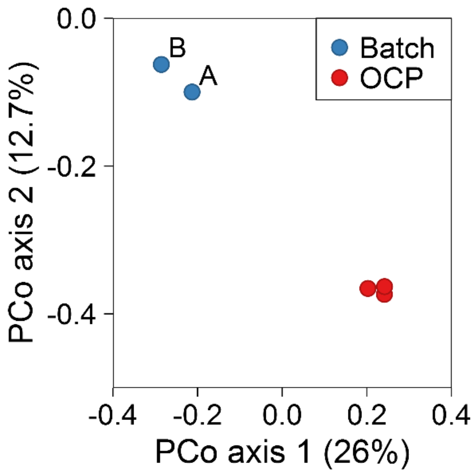

Figure S 13: Principal coordinate analysis of Unweighted Unifrac distances for microbial communities from batch-mode cells A & B and OCP electrodes A, B & C.

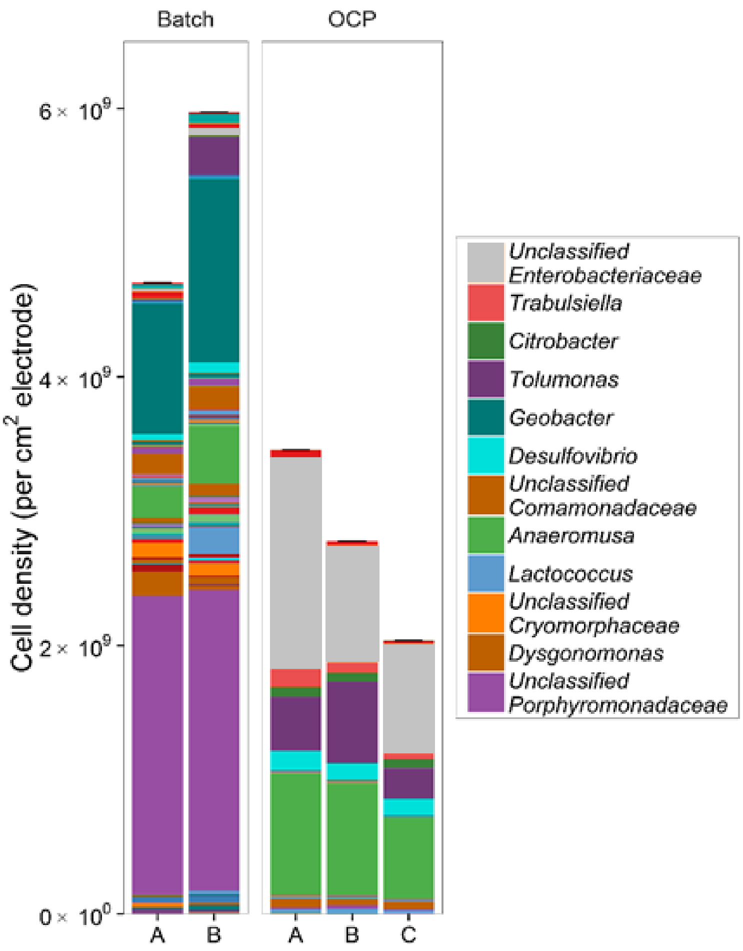

Figure S 14: Cell density weighted abundance of bacterial taxa (genus level characterization) obtained from Ion Torrent sequencing of 16S rRNA genes from batch-mode MFC anodes A and B and electrodes incubated at open circuit potential (OCP A, B and C). Error bars are the standard error from cell count procedure (black lines atop each bar).

Additional metadata are available at: http://dx.doi.org/10.25405/data.ncl.11674137.
